# Bio-Engineered Graphene-Based Cage for Efficient Local Enrichment and Biodegradation of Aqueous Organic Wastes

**DOI:** 10.1038/s41598-017-01539-0

**Published:** 2017-04-28

**Authors:** Jixiang Fan, Dongyun Chen, Najun Li, Qingfeng Xu, Hua Li, Jinghui He, Jianmei Lu

**Affiliations:** College of Chemistry, Chemical Engineering and Materials Science, Collaborative Innovation Center of Suzhou Nano Science and Technology, Soochow University, Suzhou, 215123 China

## Abstract

Microorganism immobilization has attracted great attention as a traditional method to overcome aqueous organic wastes containing N, N-dimethylformamide (DMF). In this approach, graphene oxide was modified with functional polymer firstly to obtain micro-composites material (PGO), and then the prepared composites were deposited on the surface of copper mesh (CM) to block the meshes and CM@PGO was achieved. Moreover, cage-shaped model was designed based on CM@PGO and *P*. *denitrificans* was packed inside the cage for batch experiments. This strategy could enrich the local concentration of DMF due to the formation of hydrogen bonds with the oxygen-containing groups from PGO and the character of bacteria in captivity could also contribute to the process of degradation. Results showed that the approach could remove DMF more efficiently about 15% compared with free microorganism and presented excellent cycling performance. Meantime, physical adsorption and chemical adsorption were both contributed to the process of PGO adsorption, and the adsorption isotherm fits Langmuir model well, furthermore, the theoretical maximum of adsorption ability calculated through Langmuir model is 95 mg/g. In other words, this cage-shaped CM@PGO provided a facile platform for treating various wastewaters by altering the species of packed microorganisms, which exhibited considerable prospects for wastewater treatment.

## Introduction

Contaminated water has become one urgent issue in the various fields of textile, plastics, chemical, pharmaceutical products, etc. Among the contaminated water, the organic pollutants have attracted significant attention in recent years, which caused serious pollution of water aspect owing to the discharge of abuse^1^. Various classes of organic pollutants were well established and investigated to harmful in different water bodies, such as, pesticides, fertilizers, phenols, hydrocarbons, plasticizers, biphenyls, dyes, detergents oils and versatile organic solvent^2, 3^. Meantime, those organic pollutants were persistent in the water because of the features of one or more cyclic ring, dearth polar functional group, various halogen substitutions and excellent miscibility^4^. Therefore, the emission abuse of pernicious organic wastes has aroused significant ecological disasters^5–7^. Thence, more effective techniques and better materials had been devoted to deal with those organic pollutants in wastewater. Over the past decades, several techniques were reported, for example, photo-catalytic^8–11^, distillation, adsorption^12^, chemical oxidation, solvent extraction and biodegradation.

Among the emerging organic pollutants, N, N-dimethylformamide (DMF) as one kind of organic pollutant drawn lots of attention because it was used widespread owing to its great miscibility with other organic solvents, good water solubility for many polymers in the fields of pesticides, pharmaceutical, and textile industries^13, 14^. However, because of the difficulty in removing thoroughly, DMF was considered to be an increasing threat to both environment and human beings^15–17^. Therefore, more effective techniques and better materials had been devoted to address DMF in wastewater. Biodegradation had been considered to be the most sustainable and economical approach^18–23^. In this context, various biodegradation processes had been proposed to remove DMF. Japanese patent publications and applications disclose the use of microorganisms to decompose DMF, such as *Micrococcus*, *Mycobacterium*, *Pseudomonas*, *Paracoccus*, *Xanthobacter*, and photosynthetic bacteria^24^. It was also reported that some types of microorganisms could decompose organic wastewater to carbon dioxide under the conditions of aerobic or anaerobic with less secondary pollution when compared with chemical methods^25^. At the same time, microorganism immobilization had turn into an engineering process owing to the advantages for increasing the stability of microbial cells, preventing the biomass-liquid outflow and dis-continuous process in operation and protecting the bacteria from high DMF concentrations as well as making the detach and reutilization of the biomass more easier^26^. Furthermore, immobilized matrix which showed excellent performance in organic pollutants enrichment may benefit to the wastewater treatment. However, the application of microorganism immobilization encountered a bottleneck owing to some disadvantages, including the low utilization of duplicate, high selectivity for immobilization carrier, difficult recovery after applying. Therefore, the carrier for microorganism immobilization played an important role in the process of biodegradation^27^. In this regard, various commercially available and regenerated carriers had attracted great attention, such as inorganic absorption materials and nano-materials^28^. As one of the most important derivatives of carbon, graphene oxide (GO) is characterized by a layered structure with oxygen functional groups bearing on the basal planes and edges^29–31^. Furthermore, GO could be used as an excellent adsorbent for wastewater treatment due to its extraordinary mechanical strength, significant specific area and unique two-dimensional structure^32, 33^. Moreover, we could improve its adsorption property by decorating functional polymers on the GO surface^34–39^. Meantime, GO coated on the matrix was also study to prevent the outflow of nanomaterial. Among those matrixes, copper mesh (CM) always as the major choice owing to the merits of modified functional group, favorable mechanical behavior, appropriate meshes and low cost compared with other matrix. However, GO only could achieve a maximal equilibrium adsorption amount of DMF instead of removing DMF completely.

**Figure 1:**
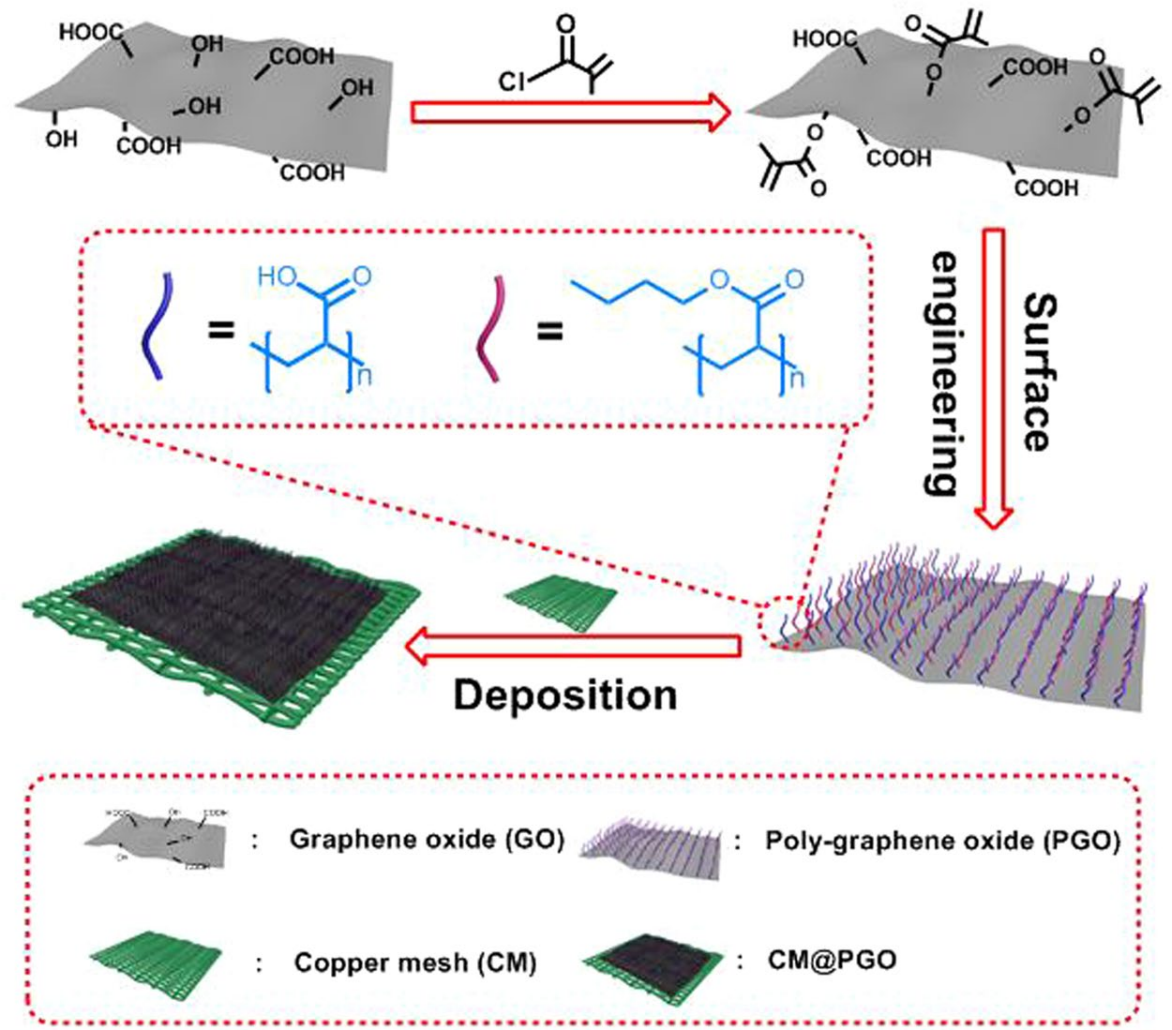
Schematic illustration of CM@PGO synthetic procedure for enriching the local concentration of DMF.

**Figure 2:**
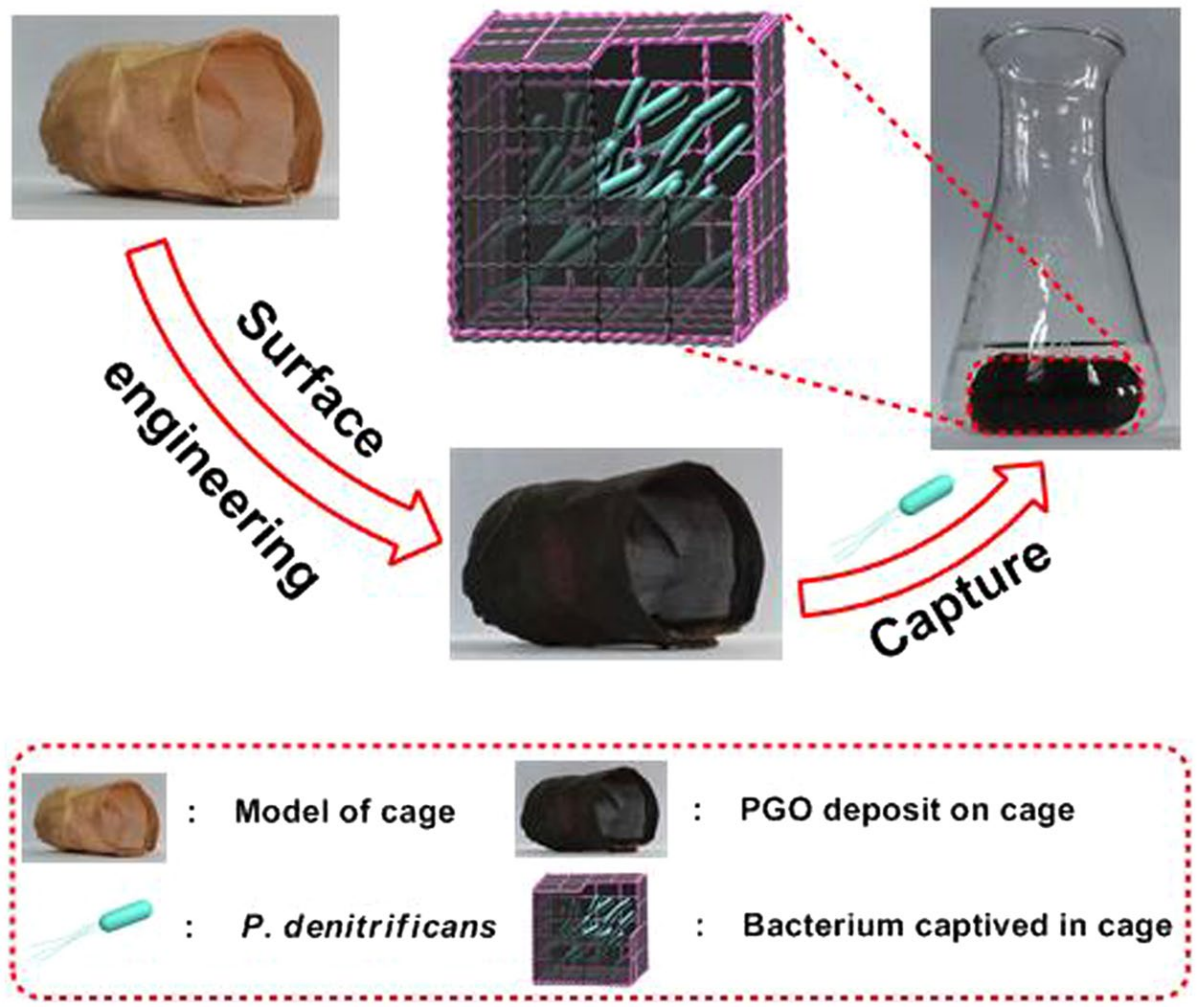
Schematic illustration of *P*. *denitrificans* captived into the cage of CM@PGO.

Herein, in order to deal with the problems mentioned above. GO was modified with meth acrylic acid (MAA) and butyl methacrylate (BMA) through free radical solution polymerization leading to one kind of excellent micro-composite material (PGO), and then the prepared composites were deposited on the surface of CM to block the meshes and CM@PGO was achieved (Fig. 1)^40^. Cage-shaped model was designed based on CM@PGO and *P*. *denitrificans* was packed inside the cage for batch experiments (Fig. 2). The strategy could enrich the local concentration of DMF because of the formation of hydrogen bond between DMF molecules and PGO. Moreover, the character of bacteria in captivity could also contribute to the process of degradation. On one hand, this approach could enrich the local concentration of DMF; on the other hand, this strategy possessed straightforward method, low cost, good circulation effect, extraordinary mechanical strength and high efficiency of biodegradation. Therefore, this unique strategy with considerable prospects could be promising candidate for the future wastewater treatment.

## Results and Discussion

**Figure 3:**
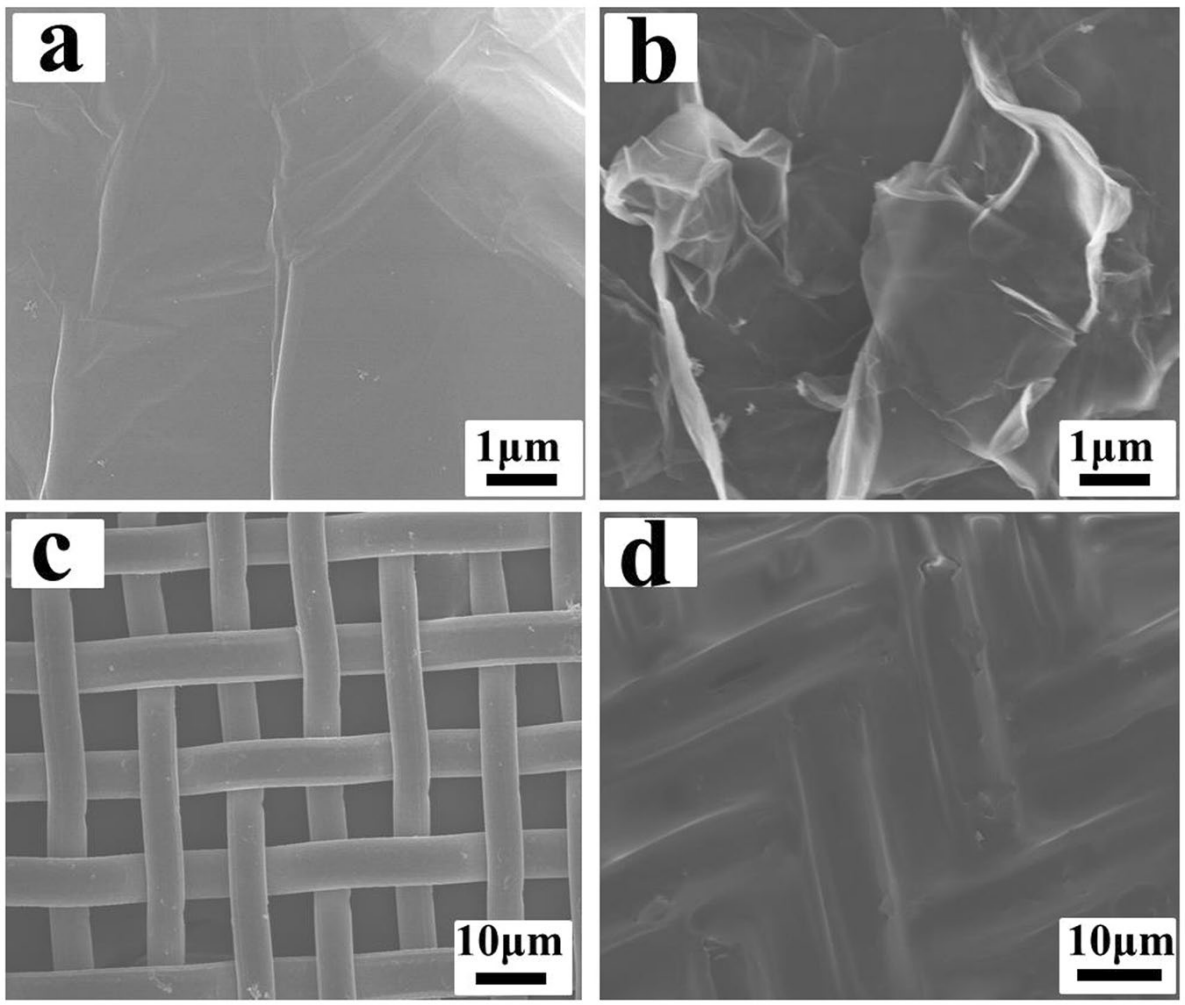
The SEM images of GO (**a**), PGO (**b**), CM (**c**) and CM@PGO (**d**).

Scanning electron microscope (SEM) photographs of the GO and PGO were shown in Fig. 3. The morphology and structure of GO was sheet and smooth. However, the morphology of PGO was rough significantly compared with GO. This suggested that the polymers were grafted on the surface of GO successfully, furthermore, the rough surface of PGO was confirmed that the PGO could be coated on the copper mesh model easier than GO. The morphology of CM@PGO (Fig. 3d) indicated that PGO blocked the copper mesh successfully and it was the condition for the next step.

**Figure 4:**
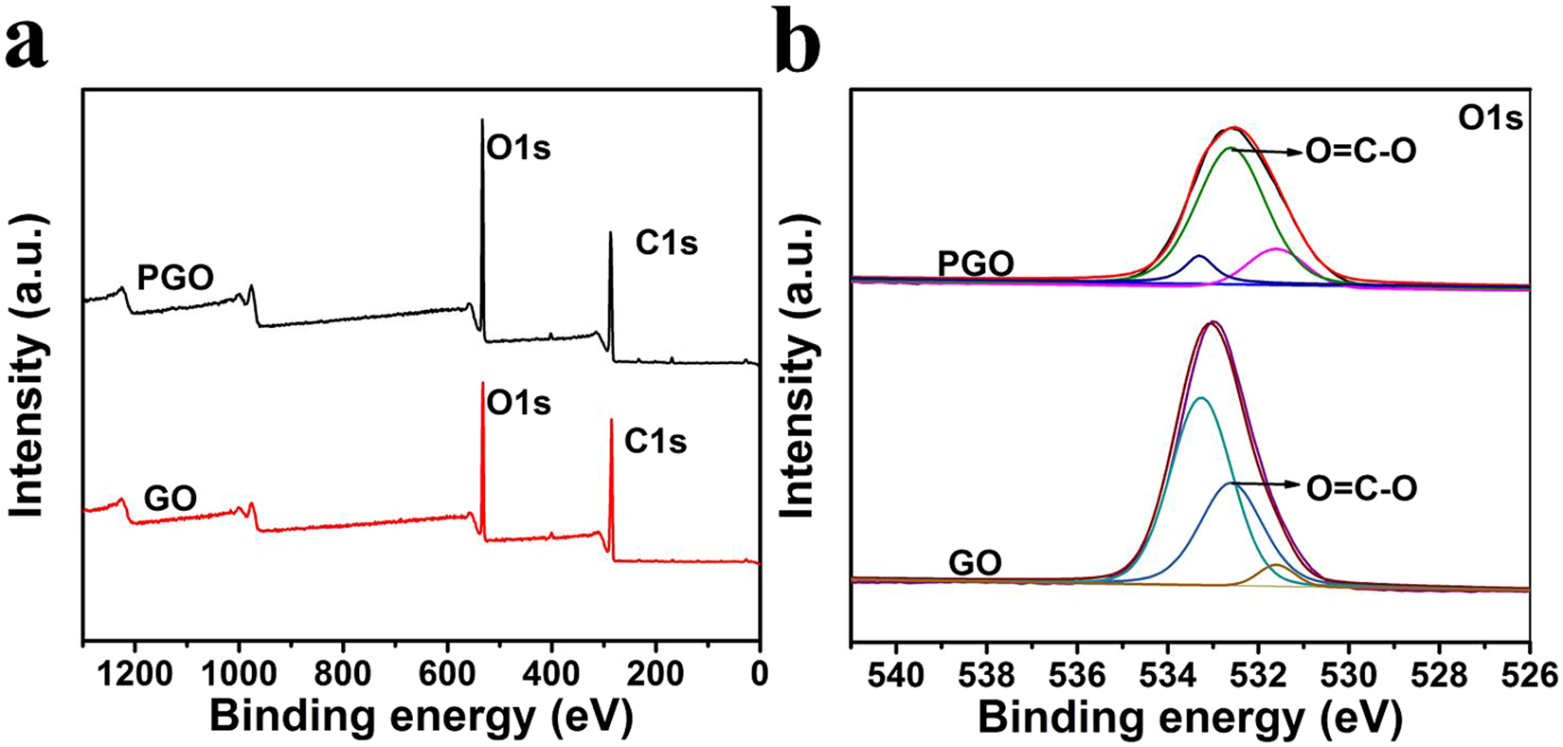
XPS survey of GO and PGO (**a**). O1s XPS of GO and PGO (**b**).

The XPS of GO and PGO were shown in Fig. 4. The spectra of O1s of GO and PGO could be fitted into three peaks for oxygen atoms in different functional groups. The peak intensity of O=C-O showed a significant increase after polymerization when compared with GO, indicating the polymer was successfully grafted on the GO.

**Figure 5:**
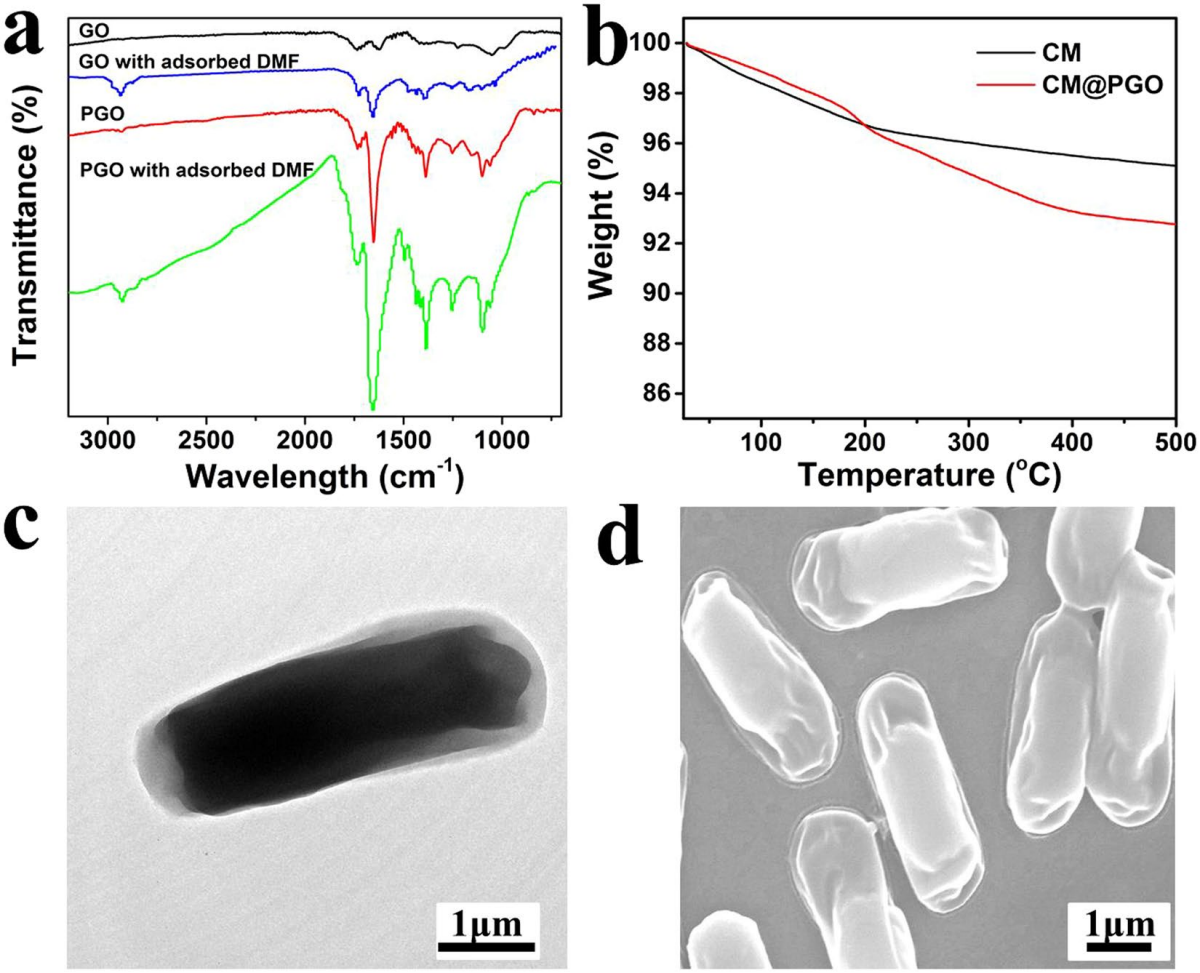
The Infrared Spectroscopy of GO, PGO and the GO, PGO with adsorbed DMF (**a**) and TGA curves of CM and CM@PGO obtained in N_2_ atmosphere with the rate of 10 °C/min (**b**) and the TEM (**c**) and SEM (**d**) images of *P*. *denitrificans*.

The model of copper mesh was fabricated and shown in Fig. 2. Dense and homogeneous layer of PGO was coated on the surface of copper mesh. At the same time, we could notice that the pore of copper mesh (5 μm shown in Fig. 3c) was blocked uniformly from the SEM of CM@PGO (Fig. 3d). Therefore, the CM@PGO was prepared successfully and the microorganism could be packed into the cage of CM@PGO. At the same time, the microorganism could not pass through the modified model due to the larger size of microorganism (1 μm shown in Fig. 5c,d) than the pores of CM@PGO (far below to achieve 1 μm investigated by Fig. 3d) and the the filtration efficiency of CM@PGO was shown in the following expermients, while the small molecules of DMF (molecular level below 1 nm) could go through the interface easily under external water pressure, which can also be investigated by *chem*. *commun*. *50*, *5586–5589* (*2014*).

**Figure 6:**
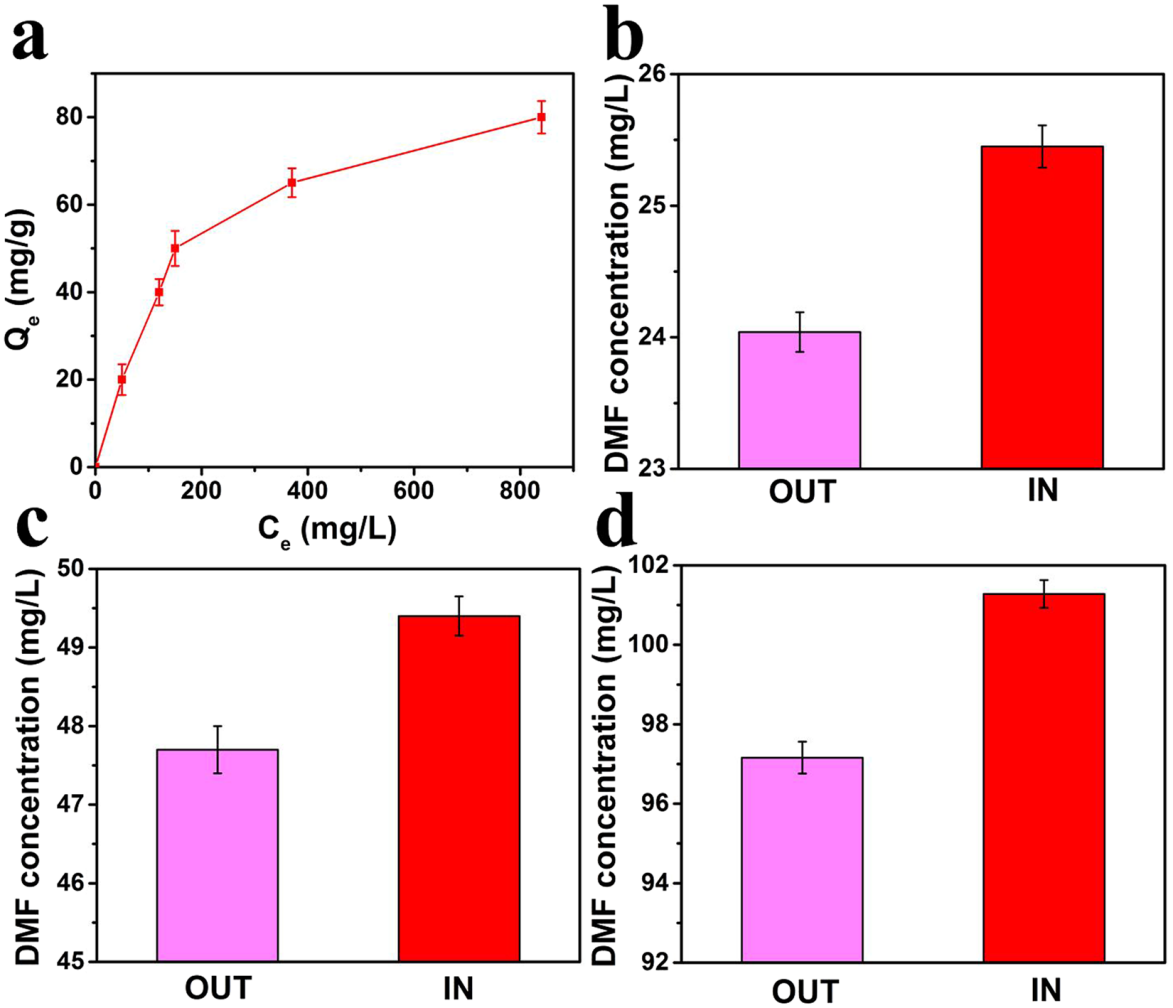
The adsorption isotherms of PGO (**a**) and the difference of DMF concentration for inside and outside of CM@PGO at the initial concentration = 25 mg/L (**b**), 50 mg/L (**c**), 100 mg/L (**d**) (DMF solution volume = 50 mL and T = 30 °C).

The adsorption spectra of GO, PGO and the GO, PGO with adsorbed DMF were shown in Fig. 5a. The presence of different oxygen-containing functional groups was observed and the increase of peaks at 1384 cm^−1^ indicated the strong bond of O = C-O, which revealed the polymers were grafted on the GO successfully. Meantime, it displayed other adsorption peak at 2930 cm^−1^ after the adsorption of DMF, which could be attributed to the asymmetric stretching vibration and symmetrical stretching vibration of CH_3_ from DMF_._ The results investigated that DMF adsorbed on GO and PGO successfully. As shown in Fig. 5b, the loading capacity of PGO on the surface of CM was almost 4% after coating 10 h, which was enough to block the mesh of CM (5 μm shown in Fig. 3c) and the result indicated that the mesh of CM was blocked completely and uniformly. (Figure 3d) and Fig. 5c,d showed the TEM and SEM of bacteria after a series of post-processing (fixed morphology by glutaraldehyde, ethanol dehydration with different concentration and freeze-dried at −40 °C for 6 h finally). Therefore, the change of morphology is possible and normal. To determine the DMF concentrations inside and outside of CM@PGO, a series of experiments were carried out and the results were shown in Fig. (6b,c and d). We could notice that the concentration inside was about 1–2 mg/L higher than outside. At the same time, it was also observed that the concentration inside was higher than initial concentration. Therefore, the model of CM@PGO could enrich local concentration of DMF as a result of polymer possessing more Van der Waals forces interaction with DMF than the single material. As shown in Fig. 6a, the adsorption ability of PGO increase with the increase of equilibrium concentration and the slope of adsorption isotherm drop gradually.

To further investigate the adsorption capacity and adsorption equilibrium of PGO, the classic models of Langmuir and Freundlich were studied. The mathematical formulas can be expressed as following:

1 $${\rm{Langmuir}}\,{\rm{model}}:{C}_{{\rm{e}}}/{Q}_{{\rm{e}}}=1/{K}_{{\rm{L}}}{Q}_{{\rm{m}}}+{C}_{{\rm{e}}}/{Q}_{{\rm{m}}}$$

2 $${\rm{Freundlich}}\,{\rm{model}}:\,\mathrm{ln}\,{Q}_{e}=\,\mathrm{ln}\,{K}_{{\rm{f}}}+1/{\rm{n}}\,\mathrm{ln}\,{C}_{{\rm{e}}}$$

Where *C*_e_ (mg/L) is the equilibrium concentration of DMF, *Q*_e_ (mg/g) is the adsorption capacity of DMF at equilibrium, *Q*_m_ is the theoretical maximum adsorption ability (mg/g), and *K*_L_, *K*_f_, are the adsorption constants of Langmuir, Freundlich, and *n* is the Freundlich linearity index.

The parameters of adsorption isotherm were presented in Table 1. The regression coefficient (*R* ^*2*^) of Langmuir (0.984) was higher than Freundlich (0.909). Therefore, the Langmuir model fits well, which indicated that the process of adsorption work on the specific homogenous surface of PGO with the same distribution of energy class and the monolayer molecule adsorption is the key during the process.

**Table 1:** Langmuir and Freundlich isotherm parameters of DMF molecules onto PGO.

T (K)	Langmuir model			Freundlich model		
	*Q*m (mg/g)	*K*L (L/mg)	*R* ^2^	*K*_F_	*n*	*R* ^2^
303	95.033	0.00628	0.984	6.502	2.639	0.909

To confirm the adsorption kinetic mechanisms of PGO, the pseudo-first-order kinetic model and pseudo-second-order kinetic model were investigated^41, 42^. The pseudo-first-order kinetic model was used for low concentration of solute. It was investigated by following equation:

3 $$\mathrm{ln}({q}_{e}-{q}_{t})=\,\mathrm{ln}\,{q}_{e}-{k}_{1}t$$

Which could be converted into:

4 $${q}_{t}={q}_{e}(1-\exp (-{k}_{1}t))$$

Where *q*_*t*_ was the DMF amount adsorbed at time *t* (mg/g); *q*_*e*_ was that adsorbed at the equilibrium (mg/g); *k*_*1*_ was the rate constant of pseudo-first-order kinetic model (min^−1^). The pseudo-second-order equation was more suitable for the amount of the solute adsorbed on the surface of adsorbent and the dosage adsorbed at equilibrium, which could be written as follows:

5 $$t/{q}_{t}=1/({k}_{2}{q}_{e}^{2})+t/{q}_{e}$$

Which could be rearranged as:

6 $${q}_{t}={k}_{2}{q}_{e}^{2}t/(1+{k}_{2}{q}_{e}t)$$

Where *k*_*2*_ was the rate constant of pseudo-second-order equation, and *t*, *q*_*e*_, and *q*_*t*_ had the same meanings as those in the pseudo-first-order equation. The data of adsorption kinetic were obtained in Table 2. We could notice that the correlation coefficient (R^2^) in pseudo-first-order model and pseudo-second-order model were both closer to 1.0. Therefore, we could conclude that the physical adsorption (came from GO) and the chemical adsorption (came from the Van der Waals forces between the DMF and polymer) were both contribute to the process of adsorption.

**Table 2:** Kinetics data of pseudo-first-order and pseudo-second-order model for the adsorption of PGO.

Pseudo-first-order [*q*_*t*_ = q_e_(1 − exp(-*k*_*1*_ *t*))]			Pseudo-second-order [*t/q*_*t*_ = 1/(*k*_*2*_ *q*_*e*_^2^) + *t*/*q*_*e*_]		
*q*_*e*_ (mg/g)	*k*_*1*_ (min^−1^)	R^2^	*q*_*e*_ (mg/g)	*k*_*2*_(g/(mg.min^−1^))	R^2^
68.565	0.00604	0.99886	86.638	7.04e-5	0.99567

**Figure 7:**
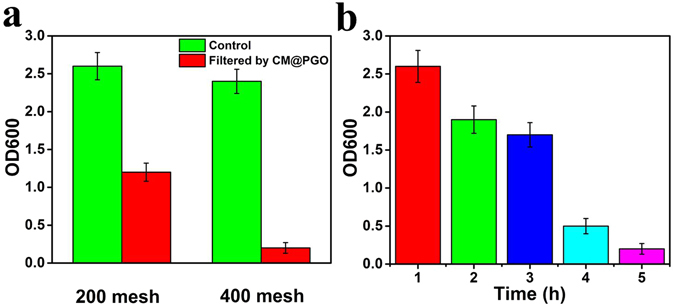
The filtration efficiency of CM@PGO with different copper mesh (**a**). The effect of coating time on OD600 (**b**).

To determine the filtration efficiency of CM@PGO, batch experiments were carried out. Figure 7a showed that bacterial content of filtrate through copper mesh with 400 mesh was lower compared with copper mesh with 200 mesh. At the same time, microbiological content of filtrate was lower and lower with the increase of the number of coating time shown in Fig. 7b. Therefore, the following experiments were carried out under the conditions of coating 10 h with 400 mesh.

**Figure 8:**
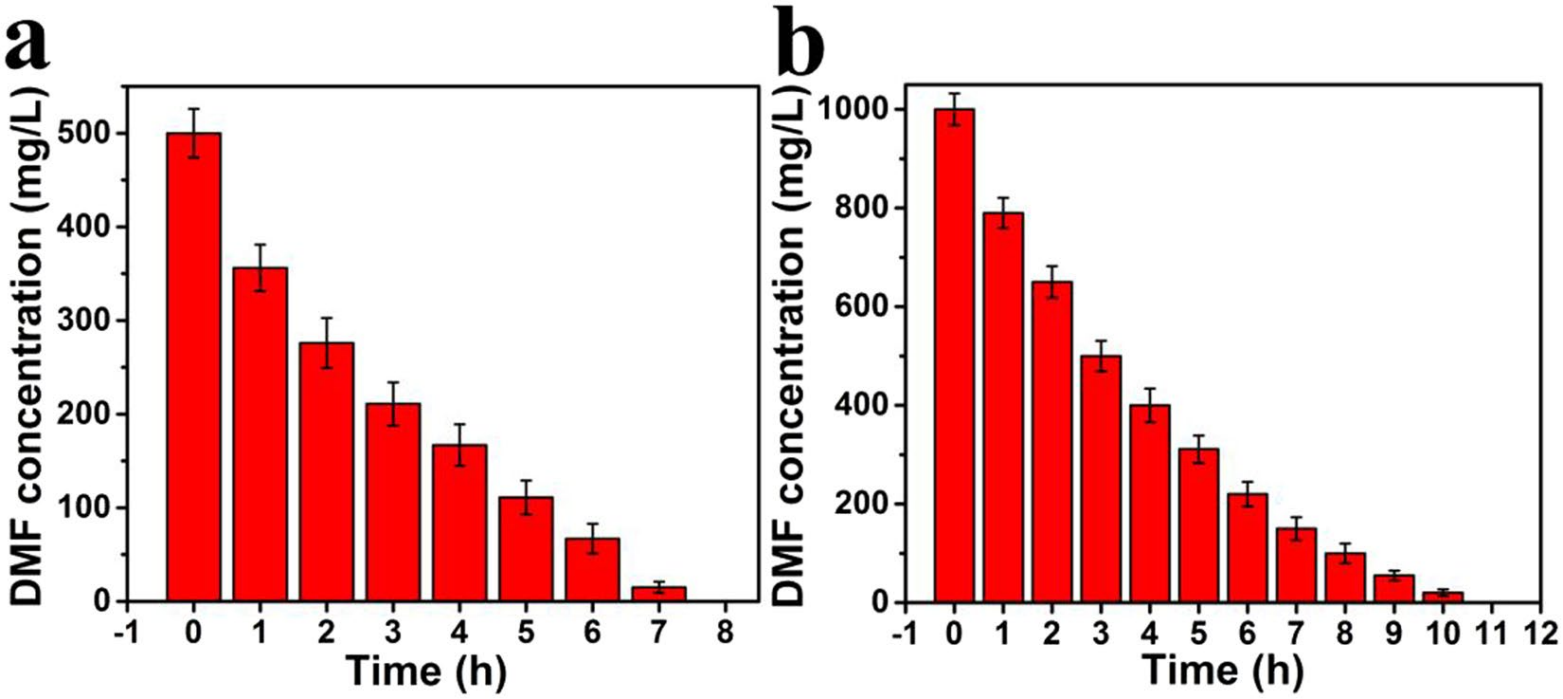
Biodegradation curve of free *P*. *denitrificans* (initial DMF concentration = 500 mg/L (**a**), 1000 mg/L (**b**), DMF solution volume = 50 mL, T = 30 °C, the weight of *P*. *denitrificans* = 2.0 g).

**Figure 9:**
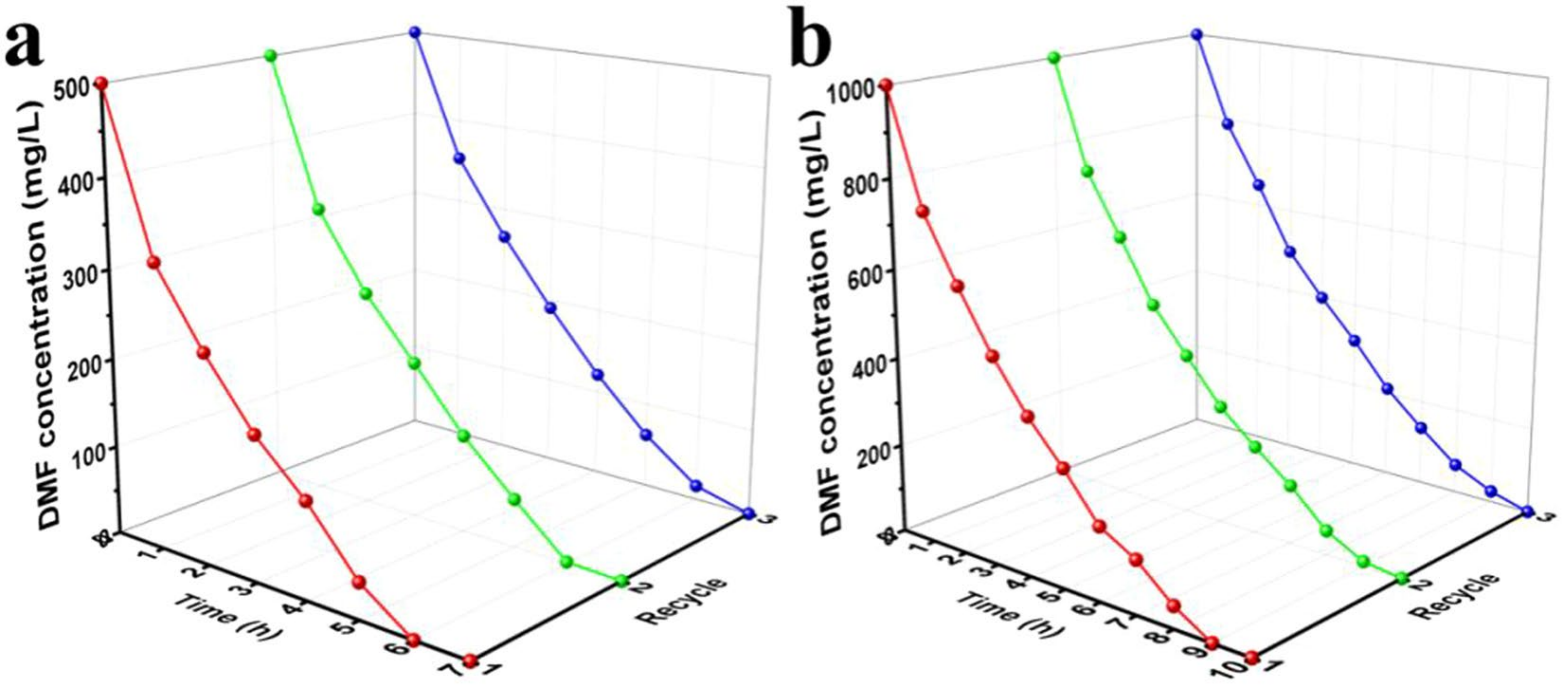
The biodegradation process of *P*. *denitrificans* packed inside cage and cycling performance (initial concentration of DMF = 500 mg/L (**a**), 1000 mg/L (**b**), the weight of *P*. *denitrificans* = 2.0 g, DMF solution volume = 50 mL, T = 30 °C).

Figures 8 and 9 showed the degradation efficiency and cycling performance of free cells and cells packed into cage. DMF was removed completely within 8 h and 11 h with initial concentration of 500 mg/L and 1000 mg/L (Fig. 8a,b). However, microorganism packed into CM@PGO could increase the efficiency of DMF about 15% compared with free cells and it was no obvious decrease in the process of biodegradation after cycling for three times (Fig. 9), because the nano-composites could raise local concentration around microorganism owing to the character of PGO adsorption and make bacteria catch the DMF molecular more easier under low concentration, meantime, microorganism packed into CM@PGO can also enhance the collision probability between DMF molecular and microorganism. And the change of inside and outside concentration of CM@PGO can be investigated by Fig. 6. These results suggested that the CM@PGO could be successfully applied for the removal of DMF from aqueous solution.

**Figure 10:**
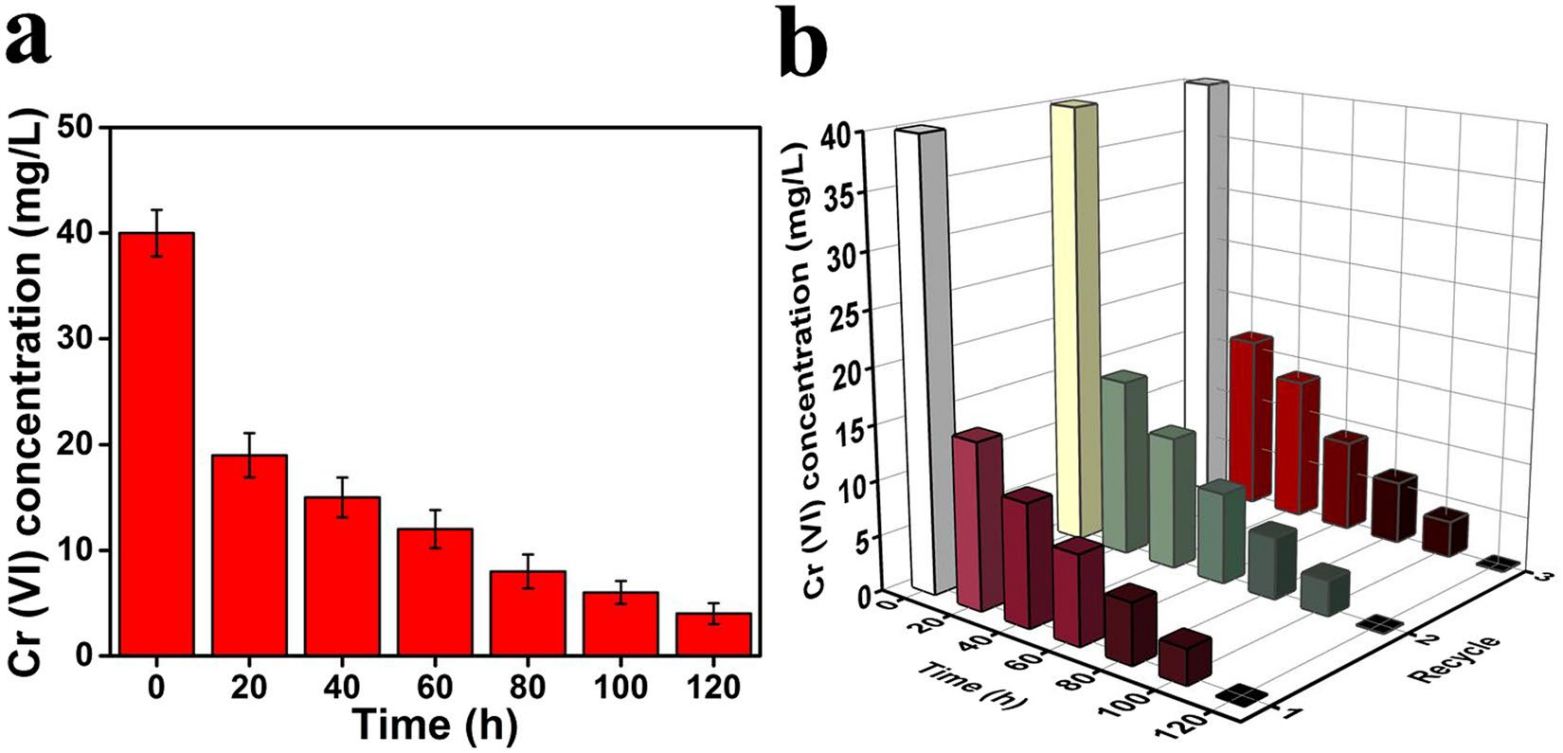
The biodegradation process of free *B*. *subtilis* (**a**) and *B*. *subtilis* packed into CM@PGO with cycling performance (**b**) (the weight of *B*. *subtilis* = 2.0 g, Cr (VI) solution volume = 50 mL, T = 30 °C).

To confirm the ascendancy of the bacterial captured into CM@PGO compared with free cells, batch experiments were carried out using *B*. *subtilis* as a model microorganism. Figure 10 showed that the degradation efficiency and cycling performance of free *B*. *subtilis* (had been investigated to degrade Cr (VI)) and *B*. *subtilis* captive into cage (Cr (VI) had no Van der Waals forces with PGO)^43^. We noticed that Cr (VI) with concentration of 40 mg/L by model could be removed within 120 h and could enhance the efficiency about 8% in comparison with free *B*. *subtilis* due to the character of *B*. *subtilis* in captivity and the cycling performance was applicable. This result indicated that the model of CM@PGO could also be applicable when microorganism was changed. Moreover, we could conclude that the enrichment of microorganism was more efficiency than the free bacteria.

## Conclusions

In summary, P. *denitrificans* was packed inside copper mesh modified with micro-composites material (PGO) depositing on the surface of CM for the efficient removal of DMF. The enrichment effects due to the formation of hydrogen bonds and the bacteria in captivity are both contribute to the enhancement of the biodegradation process. Results showed that the approach could remove DMF more efficiently about 15% compared with free bacteria and performed excellent cycling performance. Meantime, physical adsorption and chemical adsorption were both contributed to the process of PGO adsorption, and the adsorption isotherm fits Langmuir model well, furthermore, the theoretical maximum of adsorption capability calculated through Langmuir model is 95 mg/g. Therefore, the strategy provided a facile platform for treating various wastewaters and exhibited considerable prospects for wastewater treatment.

## Methods

### Materials

The copper meshes with different mesh sizes were commercially available. Tryptone and yeast extract were commercially get from Suzhou Biogene Biotechnology Co., Ltd. Graphite flake, methacryloyl chloride (MAC), meth acrylic acid (MAA), butyl methacrylate (BMA) was purchased from Sinopharm Chemical Reagent Co., Ltd, and can be used directly without no further processing. HPLC grade methanol was purchased from Shanghai Qiangshun Chemical Reagent Co., Ltd.

### Synthesis of GO

GO was synthesized through modified Hummers method^44^. In short, graphite flake (1.5 g) was added to a 9:1 mixture of concentrated H_2_SO_4_/H_3_PO_4_ (180:20 mL) slowly, made sure the temperature less than 40 °C. Then, this reaction was heated to 50 °C and stirred for 12 h. The mixture was poured onto ice (200 mL) when the reaction was cooled to Rt. Next, 30% H_2_O_2_ was added to the mixture drop by drop until the color of the solution from brown turned to bright yellow. Then, there were some precipitation in bottom of the beaker overnight. The remaining solid material was centrifuged and washed with HCl (5%), water and ethanol, for each wash, the supernatant was decanted away. Subsequently, the material was dissolve in deionized water and dialyzed for one week to remove the remaining acid. Finally, the mixture was centrifuged to get concentrated liquid and dried in the vacuum desiccator. The prepared GO was used for the next step of the experiments.

### Synthesis of PGO

PGO was prepared by two steps based on a reported procedure with a slight modification^26^.

### Preparation of copper mesh model

Copper mesh was cleaned by ultrasonic for 3 h in water and acetone respectively. The copper mesh was immersed into PGO aqueous solution and ultrasonic for 10 min, then the model was dry in the vacuum desiccator at 60 °C, subsequently this model was put into PGO aqueous solution and dried in the vacuum desiccator again and again until the surface of copper mesh model was covered with a layer of dense PGO^40^.

### Filtration efficiency of CM@PGO

Two clean copper meshes with different mesh were coated with PGO aqueous solution by ultrasonic for 10 min respectively, then dried in a vacuum oven at 50 °C. After dried for each time, draw some *P*. *denitrificans* (had been grew into the logarithmic phased) and determined the OD600 of filtrate after dripping on copper mesh.

### Uptake capacity of PGO

The experiments of adsorption capacity were measured in 250 mL Erlenmeyer flask in the constant temperature oscillator at 30 °C for 120 r/min. 100 mg PGO was added in the 50 mL aqueous solution containing different concentration of DMF respectively and sampling overtime. And adsorption capacity at equilibrium was calculated as^45^:

7 $${Q}_{e}=({C}_{0}-{C}_{e})V/M$$

Where *C*_*0*_ (mg/L) and *C*_*e*_ (mg/L) are the initial and equilibrium concentrations of DMF respectively, *m* (g) is the mass of adsorbent, and *V* (L) is solution volume.

### Concentration inside and outside of CM@PGO

The model of CM@PGO was put into DMF aqueous solution containing with concentration of 25 mg/L, 50 mg/L and 100 mg/L. Made sure that the aqueous liquid level of DMF was below on the model of CM@PGO and sampling overtime.

### Bacterial culture

Luria Bertani (LB) liquid medium (Tryptone, 10 g/L; Yeast extract, 5 g/L; NaCl, 10 g/L), minimal salts medium (MM1) solution (K_2_HPO_4_, 6.3 g/L; KH_2_PO_4_, 1.8 g/L; MgSO_4_·7H_2_O, 0.1 g/L; MnSO_4_·4H_2_O, 0.1 g/L; CaCl_2_·2H_2_O, 0.1 g/L; FeSO_4_·7H_2_O, 0.1 g/L; NaMoO_7_·7H_2_O, 0.006 g/L) and instrument were disinfected at 121 °C for 20 min, the mother liquor of DMF was sterilized by 0.22 μm membrane filter before using. A pure strain of *P*. *denitrificans* (ATCC 19367) was purchased from Shanghai Fuxiang Biotechnology Co. Ltd. The bacterium after acclimatization was cultured through LB, incubated at 30 °C and 150 r/min on the constant temperature oscillator (SHA-CA).

### Degradation experiments

The optimal conditions of the biodegradation by bacteria were reported before^26^. Then, the experiment of degradation was carried out at 30 °C on the constant temperature oscillator at 150 r/min. After culturing, the bacteria was centrifuged and washed with PBS solution for three times. Subsequently 2 g bacteria (wet weight) was dispersed into MM1 solution (50 mL) containing 500 mg/L (1000 mg/L) DMF in 100 mL Erlenmeyer flasks. At the same time, the same wet weight of bacteria was put into the model of CM@PGO. Finally, the system was cultivated on the constant temperature oscillator and sampling every hour.

### Analytical methods

The concentration of DMF was measured by high performance liquid chromatograph (HPLC, Agilent 1260 Infinity) using an Accurasil C18 column (4.6 mm * 150 mm inner diameter, 5 mm particle size) at 25 °C, G1311C pumps and Ultraviolet detector (1260 MWD) at 205 nm. The proportion of mobile phase is methanol and water (methanol: water = 15: 85) and velocity of mobile phase is 1 mL/min. The thermal stability of GO and PGO was monitored through TG209F1 Libra thermal analyzerat under the condition of N_2_ atmosphere and the temperature was raised to 650 °C from 30 °C at the rate of 10 °C/min. The content of C-C bond was measured by XPS (ESCALAB 250XI). Vibrations of chemical bonds for GO and PGO was recorded by Fourier transform infrared spectroscopy (Nicolet 4700). The morphology of product was analyzed by SEM (Hitachi S-4800). The OD600 value of bacteria was collected through UV-Vis spectrophotometer (TU-1901).

## References

[1] Yang M (2011). A current global view of environmental and occupational cancers. J. Environ. Sci. Health Part. C..

[2] Ali, I. & Aboul-Enein, H. Y. Chiral pollutants: distribution, toxicity and analysis by chromatography and capillary electrophoresis. *John Wiley & Sons Chichester* (2004).

[3] Damià, B. *et al*. Emerging organic pollutants in waste waters and sludge. Springer Berlin (2005).

[4] Lövblad, G., Tarrasón, L., Tørseth, K. *et al*. EMEP assessment part I: European perspective. *Norwegian Meteorological Institute*, *PO Box*. **43** (2004).

[5] Podila R, Brown JM (2013). Toxicity of engineered nanomaterials: a physicochemical perspective. J. Biochem. Mol. Toxic.

[6] Chen P-C, Xu Z-K (2013). Mineral-coated polymer membranes with superhydrophilicity and underwater superoleophobicity for effective oil/water separation. Sci. Rep..

[7] Jin M, Wang J, Yao X, Liao M, Zhao Y, Jiang L (2011). Underwater Oil Capture by a Three‐Dimensional Network Architectured Organosilane Surface. Adv. Mater..

[8] Kumar A (2014). Polyacrylamide/Ni_0.02_Zn_0.98_O nanocomposite with high solar light photocatalytic activity and efficient adsorption capacity for toxic dye removal. Ind. Eng. Chem. Res..

[9] Kumar A (2015). SPION/β-cyclodextrin core–shell nanostructures for oil spill remediation and organic pollutant removal from waste water. Chem. Eng. J..

[10] Kumar A (2016). Magnetically recoverable ZrO_2_/Fe_3_O_4_/chitosan nano-materials for enhanced sunlight driven photoreduction of carcinogenic Cr (VI) and dechlorination & mineralization of 4-chlorophenol from simulated waste water. RSC. Adv..

[11] Pathania D, Sharma G, Kumar A (2015). Combined sorptional-photo-catalytic remediation of dyes by polyaniline Zr(IV) selenotungstophosphate nanocomposite. Toxicol. Environ. Chem..

[12] Naushad M, Ahamad T, Sharma G (2016). Synthesis and characterization of a new starch/SnO_2_ nanocomposite for efficient adsorption of toxic Hg^2+^ metal ion. Chem. Eng. J..

[13] Redlich CA (1988). Liver disease associated with occupational exposure to the solvent dimethylformamide. Ann. Intern. Med..

[14] Ghisalba O, KuÈenzi M, Schär H-P (1986). Biodegradation and utilization of N, N-dimethylformamide by specialized methylotrophs. Cell. Mol. Life Sci..

[15] Käfferlein HU, Angerer J (2001). N-methylcarbamoylated valine of hemoglobin in humans after exposure to N, N-dimethylformamide: evidence for the formation of methyl isocyanate. Chem. Res. Toxicol..

[16] Shieh D-B (2007). Mitochondrial DNA alterations in blood of the humans exposed to N, N-dimethylformamide. Chem-Biol. Interact..

[17] Kim TH (2010). Synergistic hepatotoxicity of N, N-dimethylformamide with carbon tetrachloride in association with endoplasmic reticulum stress. Chem-Biol. Interact..

[18] Kumar SS, Kumar MS, Siddavattam D, Karegoudar T (2012). Generation of continuous packed bed reactor with PVA–alginate blend immobilized Ochrobactrum sp. DGVK1 cells for effective removal of N, N-dimethylformamide from industrial effluents. J. Hazard. Mater..

[19] Vidhya R, Thatheyus A (2013). Biodegradation of dimethylformamide using Bacillus subtilis. Am. J. Microbiol. Res..

[20] Swaroop S, Sughosh P, Ramanathan G (2009). Biomineralization of N, N-dimethylformamide by Paracoccus sp. strain DMF. J. Hazard. Mater..

[21] Sanjeevkumar S, Nayak AS, Santoshkumar M, Siddavattam D, Karegoudar T (2013). Paracoccus denitrificans SD1 mediated augmentation with indigenous mixed cultures for enhanced removal of N, N-dimethylformamide from industrial effluents. Biochem. Eng. J.

[22] Veeranagouda Y, Paul PE, Gorla P, Siddavattam D, Karegoudar T (2006). Complete mineralisation of dimethylformamide by Ochrobactrum sp. DGVK1 isolated from the soil samples collected from the coalmine leftovers. Appl. Microbiol. Biot..

[23] Yang N, Chen X, Lin F, Ding Y, Zhao J (2014). Toxicity formation and distribution in activated sludge during treatment of N, N-dimethylformamide (DMF) wastewater. J. Hazard. Mater..

[24] Okazaki. (1995). Development of poly (vinyl alcohol) hydrogel for waste water cleaning. II. Treatment of N, N‐dimethylformamide in waste water with poly (vinyl alcohol) gel with immobilized microorganisms. J. Appl. Polym. Sci..

[25] Bai J, Wen J-P, Li H-M, Jiang Y (2007). Kinetic modeling of growth and biodegradation of phenol and m-cresol using Alcaligenes faecalis. Process Biochem..

[26] Zheng Y (2016). Efficient simultaneous adsorption-biodegradation of high-concentrated N, N-dimethylformamide from water by Paracoccus denitrificans-graphene oxide microcomposites. Sci. Rep..

[27] Matsui Y (2015). Adsorption capacities of activated carbons for geosmin and 2-methylisoborneol vary with activated carbon particle size: Effects of adsorbent and adsorbate characteristics. Water Res..

[28] Khin MM, Nair AS, Babu VJ, Murugan R, Ramakrishna S (2012). A review on nanomaterials for environmental remediation. Energ. Environ. Sci..

[29] Lerf A, He H, Forster M, Klinowski J (1998). Structure of graphite oxide revisited. J. Phys. Chem. B..

[30] Stankovich S, Piner RD, Nguyen ST, Ruoff RS (2006). Synthesis and exfoliation of isocyanate-treated graphene oxide nanoplatelets. Carbon.

[31] Jeong H-K (2008). Evidence of graphitic AB stacking order of graphite oxides. J. Am. Chem. Soc..

[32] Geim AK, Novoselov KS (2007). The rise of graphene. Nat. Mater..

[33] Park S, Ruoff RS (2009). Chemical methods for the production of graphenes. Nat. Nanotechnol..

[34] Chen Y, Chen L, Bai H, Li L (2013). Graphene oxide–chitosan composite hydrogels as broad-spectrum adsorbents for water purification. J. Mater. Chem. A..

[35] Sui Z-Y, Cui Y, Zhu J-H, Han B-H (2013). Preparation of three-dimensional graphene oxide–polyethylenimine porous materials as dye and gas adsorbents. Appl. Mater. Inter..

[36] Shi H, Li W, Zhong L, Xu C (2014). Methylene blue adsorption from aqueous solution by magnetic cellulose/graphene oxide composite: Equilibrium, kinetics, and thermodynamics. Ind. Eng. Chem. Res..

[37] Wu Z (2014). Adsorptive removal of methylene blue by rhamnolipid-functionalized graphene oxide from wastewater. Water Res..

[38] Shen Y, Chen B (2015). Sulfonated graphene nanosheets as a superb adsorbent for various environmental pollutants in water. Environ. Sci. Technol..

[39] Pastrana-Martínez LM, Morales-Torres S, Figueiredo JL, Faria JL, Silva AM (2015). Graphene oxide based ultrafiltration membranes for photocatalytic degradation of organic pollutants in salty water. Water Res..

[40] Dong Y, Li J, Shi L, Wang X, Guo Z, Liu W (2014). Underwater superoleophobic graphene oxide coated meshes for the separation of oil and water. Chem. Commun..

[41] Park Y (2013). Structural characterisation and environmental application of organoclays for the removal of phenolic compounds. J. Colloid. Interf. Sci..

[42] Ho Y-S, McKay G (1999). Pseudo-second order model for sorption processes. Process Biochem..

[43] Pan X (2014). Investigation of Cr(VI) reduction and Cr(III) immobilization mechanism by planktonic cells and biofilms of Bacillus subtilis ATCC-6633. Water Res..

[44] Marcano DC (2010). Improved synthesis of graphene oxide. ACS nano.

[45] Zhang H-K, Lu H, Wang J, Zhou J-T, Sui M (2014). Cr (VI) reduction and Cr (III) immobilization by Acinetobacter sp. HK-1 with the assistance of a novel quinone/graphene oxide composite. Environ. Sci. Technol..

